# Comparison of the University Pharmacy Education Programs in Germany and Vietnam

**DOI:** 10.3390/pharmacy10060146

**Published:** 2022-11-02

**Authors:** Daniel Baecker, Do Thi Mai Dung, Hai Pham-The, Nguyen Hai-Nam

**Affiliations:** 1 Department of Pharmaceutical and Medicinal Chemistry, Institute of Pharmacy, University of Greifswald, Friedrich-Ludwig-Jahn-Straße 17, 17489 Greifswald, Germany; 2 Hanoi University of Pharmacy, 13-15 Le Thanh Tong, Hoan Kiem, Hanoi 10000, Vietnam

**Keywords:** pharmacy education, study program, access to studies, structure of studies, further training opportunities, Germany, Vietnam, international exchange, teaching staff mobility

## Abstract

During the global COVID pandemic, the importance of professionals in the health care sector has been put in a new light, including pharmacists. In this context, the focus is also on how pharmacists are trained in different countries. Through an exchange of pharmacy teaching staff from a German to a Vietnamese university, the pharmacy education programs in both countries were compared. Aspects such as access to studies, structure of studies, and further training opportunities were considered. Differences and similarities emerged. In both countries, students first acquire basic knowledge and then delve deeper into pharmaceutical content in main studies. There is, expectedly, a great overlap in the content of the courses. Overall, the education at Vietnamese universities seems to be more practice-oriented due to a large number of placements. This also allows a specialization, which can be pursued in Germany with self-interest after graduation. There, the preparation for everyday work in the community pharmacy is separated from the university by a mandatory practical year. For the future, efforts are being made in both countries to strengthen the importance of clinical pharmacy in the curriculum. To this end, the Vietnamese are taking their inspiration from abroad in many cases, including Germany.

## 1. Introduction

During the COVID-19 pandemic, the social necessity of health care professions became very clear, including pharmacists [1]. The latter are trained by studying at university. Especially in times with a shortage of skilled workers, training is once again being put in a new light. However, in the past, a lot of attention has always been paid to pharmacy education in different countries around the world. In the literature, there are publications related to pharmacy education in North America (e.g., Canada [2], USA [3]), the Caribbean (e.g., Cuba [4]), South America (e.g., Brazil [5]), Europe (e.g., UK [6], France [7]), Africa (e.g., Nigeria [8], Sudan [9], Zimbabwe [10]), in Middle Eastern countries (e.g., Jordan [11], Kuwait [12], Lebanon [13], Saudi Arabia [14,15], Yemen [16,17], among others [18]), South Asia (e.g., India [19,20,21], Bangladesh [22]), South East Asia (e.g., Thailand [23,24], Vietnam [25]), and Australia [26].

Within a country, the structure of the studies of pharmacy is largely uniform due to national regulations. In an international comparison, however, differences might be seen [27]. Often, these differences are based on varying conditions or even restrictions, which are due to different development progress in countries on different continents. This makes it necessary to see things differently and to rethink them in a way that one might not have thought about them oneself. Therefore, the opportunity arises to get to know each other through comparisons. This can take place, for example, within the framework of an international staff exchange. However, to the best of our knowledge, until now, there is no report that looks at the comparison of pharmacy programs in countries on two different continents.

The purpose of a teaching staff exchange was therefore to compare the systems of pharmacy studies in Germany and Vietnam, located in Europe and South East Asia, respectively. On the one hand, this generally broadens the horizon to see how things work in another country. On the other hand, by mutual comparison, one can obtain the advice about what should definitely be kept in the structure of one’s own studies. This is of particular interest to the community that is dedicated to the education of pharmacists and is involved in the design and development of curricula. Through the inspiration from the foreign country, one can even adapt or adopt things if necessary. Ideally, such an exchange will lead to a further improvement of certain parts of the pharmacy studies.

However, the aim of this perspective is first to provide an overview of the structure of pharmacy studies in the two countries of Germany and Vietnam. Surprisingly, we found that, to our best knowledge, there is no publication dealing with the topic of the general structure of pharmacy studies in Germany so far. Thus, this paper is a contribution to the literature. In contrast, although there is a work on pharmacy education in Vietnam [25], it is almost 10 years old. Since many things have changed in the meantime, we provide an update in our manuscript. In addition, the curricula in both countries were compared.

## 2. Approach

The comparison of pharmacy education was prompted by the exchange of a German pharmacy faculty member who was a guest lecturer at a Vietnamese pharmacy university. This basically included three steps. First, during the stay as a guest lecturer at the Hanoi University of Pharmacy (HUP), a workshop was held to present the structure of the pharmacy studies in Germany. On this occasion, the lecture entitled "Insights into the studies of pharmacy in Germany" took place on 2 August 2022. The lecture included, for example, an overview of the modalities of admission to pharmacy studies in Germany as well as the different locations. The main focus was on the structural organization of the studies and on courses in the individual sections of the studies in accordance with the legally prescribed training regulations. In addition to information on the major final examinations, the main fields of work as a trained pharmacist in Germany were presented. Further training and specialization opportunities after university studies were also mentioned.

The lecture was attended by a total of more than 80 participants from HUP, ranging from junior to advanced students, PhD students, postdocs, lecturers, from professors to the vice rector and the rector of the university. Thus, the view from all perspectives was covered on the Vietnamese side. After the lecture given in English, the presentation documents translated into Vietnamese were read out again in order to eliminate any language barrier and to guarantee full understanding of the transmitted contents (especially for students).

Second, there was an extensive questions and answers session with the speaker and a lively discussion on the structures of the study programs at the two locations. Based on this, it was possible to derive commonalities and differences between the education in both countries. Third, there were several formal discussions with each other during the stay, in which both the German guest lecturer and Vietnamese lecturers worked out the comparison jointly. These included, among others, an analysis of the admission procedure, the structural organization of the study program as described in the respective legal regulations, as well as the subject areas, the types of examinations, and the conditions for obtaining the professional license. In this way, a clear understanding of the training system in the respective other country was created, which in turn enabled a comparison of pharmacy studies in Germany with the BPharm degree programs in Vietnam. It should be mentioned that in the present comparison, public universities but not private universities of Vietnam (and their accreditation/approval process) are covered in this manuscript.

## 3. Presentation of Pharmacy Programs in Germany and Vietnam

One general goal of a teaching staff exchange is to get to know each other and the environment at the foreign university. Special focus is put on the teaching and education of students [28]. In order to be able to compare the study programs in the two countries of Germany and Vietnam and thus get to know each other, it is first important to introduce the particular conditions regarding the study of pharmacy in the respective country.

### 3.1. Pharmacy studies in Germany

#### 3.1.1. Admission to study

There is a nationwide admission restriction for pharmacy studies in Germany. The application for a place to study as well as the final allocation is carried out centrally by a "Foundation for University Admission" (located in the city of Dortmund). The task of this foundation is to support the universities in carrying out the procedures of application and local admission. It coordinates the allocation of study places with restricted admissions such as pharmacy but does not determine the number of pharmacy students throughout Germany (independently of this, the foundation is also responsible for the allocation of the study programs in human medicine, veterinary medicine, and dentistry). In order to obtain a study place for pharmacy in Germany, an application must be submitted to this foundation with the specific request of the study subject, i.e., pharmacy. The reason for this approach is that the number of applicants significantly exceeds the number of available study places. The central allocation is thus intended to ensure fairness throughout the country [29].

**Figure 1:**
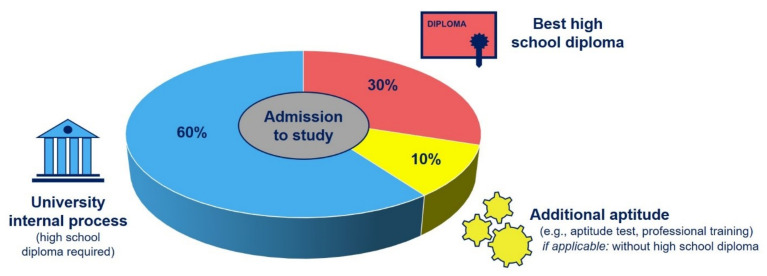
Schematic overview of the quotas of the three different admission options to pharmacy studies in Germany.

The allocation procedure can essentially be divided into three parts (see Figure 1). These cover the quota for the best high school diploma, the quota for the additional aptitude, and the quota for the requirement guidelines of the universities.

Accordingly, 30% of the study places per university are assigned to applicants who are among the best school graduates in terms of the grades in their high school diploma. A further 10% receive a study place at university according to criteria independent of school grades. This proportion has further additional skills acquired, for example, demonstrated through completed professional training or through a subject-specific aptitude test at two locations (Greifswald, Hamburg) [30]. In the latter, aspects of mathematics and physics as well as biology and chemistry are tested in the format of multiple-choice questions. Even though usually, the high school diploma is considered a basic requirement for studying at a university, the latter group can also include students without a high school diploma and allow them to study at the university as well. The majority of the study places, namely 60%, are awarded in a university-internal selection process.

In this context, it is worth mentioning that the "Foundation for University Admission" only acts as a coordinator for the respective universities. The particular universities themselves determine the guidelines for selecting applicants for a study place.

#### 3.1.2. Study Locations

In Germany, there are a total of 22 locations where pharmacy can be studied (see Table 1). In the academic year 2020/2021, a total of 16,307 pharmacy students were enrolled at the 22 campuses, which corresponds to an increase in the number of students of about 20% compared to the academic year 2011/2012 (13,603 pharmacy students) [31]. The continuous increase in the number of students can be considered as a success for the growing demand for pharmacists. It is possible to study in almost all German federal states, with two exceptions, namely Bremen and Brandenburg. Bremen (about 0.67 million inhabitants) is a federal city state and rather small compared to the other two federal city states in Germany, namely Berlin (about 3.6 million inhabitants) and Hamburg (about 1.8 million inhabitants) [32]. In addition, the state of Brandenburg does not yet have a university with pharmacy as a degree program. For years, however, there have been efforts to integrate pharmacy studies there as well at the university in the city of Cottbus-Senftenberg [33]. Apart from the size of the university, the 22 pharmacy study locations in Germany differ essentially in whether it is only possible to start studying in the winter semester or in addition in the summer semester (see Table 1). In principle, there are no tuition fees to study pharmacy in Germany at one of the locations mentioned.

#### 3.1.3. Structure of the Study

**Figure 2:**
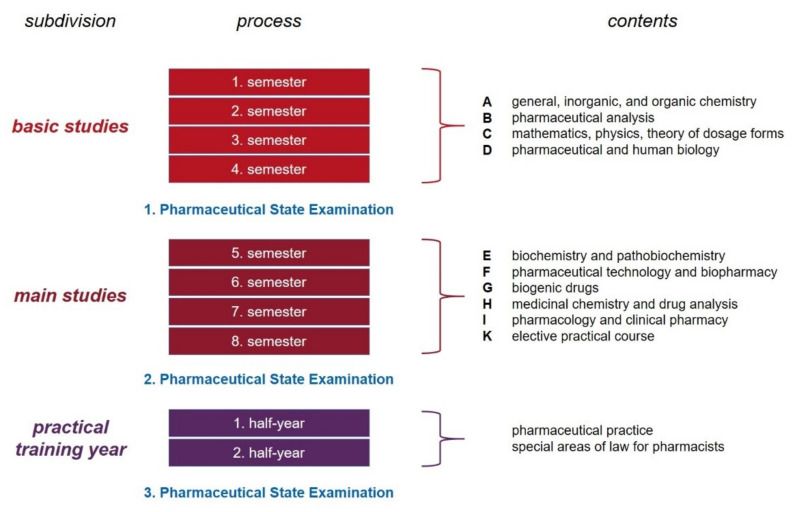
Schematic overview of the pharmacy education process in Germany.

The program of pharmacy studies is regulated throughout Germany in a statutory order, so-called “Licensing Regulations for Pharmacists” [35]. Overall, the training can be divided into three sections (see Figure 2). The mandatory standard period of study at a university comprises eight semesters. Of these, four semesters are spent on basic studies and a further four on main studies. This is followed by a non-university part to complete the full education to become a pharmacist. It lasts another two semesters. At the end of each of these three parts, graduation takes place with a state examination.

During the basic studies, the fundamental principles of natural science subjects are to be taught. The individual lectures, seminars, and laboratory practical courses are defined in the official regulations and assigned to four different subject areas (see Figure 2). Subject area A (462 teaching hours, of which 336 h are practical and 56 h are seminars) covers general, inorganic, and organic chemistry, while subject area B (392 teaching hours, of which 308 h are practical) deals with pharmaceutical analysis. The subject area C (280 teaching hours, of which 140 h are practical and 14 h are seminars) appears somewhat heterogeneous consisting of the basics of mathematics, physics, as well as theory of pharmaceutical dosage forms. Subject area D (392 teaching hours, of which 210 h are practical) is devoted to the fundamentals of pharmaceutical and human biology [35].

The completion of an 8-week practical traineeship during the lecture-free season is also obligatory in the course of the basic studies. At least four weeks must be spent in a public pharmacy. Alternatively, it is also possible to complete the other half of the eight weeks in a hospital pharmacy, in the pharmaceutical industry, or in a drug investigatory institute. The aim of the traineeship is to familiarize the students with pharmaceutical activities, organization, and operational procedures in a community pharmacy already during their basic studies. In addition, insights into legal regulations for the operation of pharmacies will be gained and technical language will be used [36].

The basic studies are completed with the first section of the pharmaceutical state examination. These are examinations that are standardized throughout Germany. They are conducted by an "Institute for Medical and Pharmaceutical Examination Questions". The examinations take place separately, but on four consecutive days in each of the four subject areas of the basic studies. The exams are written in the form of multiple-choice questions. Subject areas A and D have a total of 100 questions. For subject areas B and C, on the other hand, there are only 80 questions [37]. Overall, however, the four subject areas A–D have the same weighting among themselves. Nevertheless, the principal discussion exists that pharmacy studies are very chemistry-heavy, while medical and clinical aspects are underrepresented [38].

In the basic studies, the scientific foundations are set. This is a prerequisite and enables the student to delve even deeper into the subject matter in the subsequent period of the main studies, focusing in particular on pharmaceutical content. The type and scope of the courses are largely regulated as well and are assigned to six different subject areas. These are biochemistry and pathobiochemistry (subject area E; 196 teaching hours, of which 98 h are practical), pharmaceutical technology and biopharmacy (subject area F; 364 teaching hours, of which 196 are practical and 42 h are seminars), biogenic drugs (subject area G, comparable to pharmacognosy; 238 teaching hours, of which 84 h are practical, 42 h are seminars), medicinal chemistry and drug analysis (subject area H; 420 teaching hours, of which 280 h are practical), as well as pharmacology and clinical pharmacy (subject area I; 406 teaching hours, of which 112 h are practical and 98 h are seminars). In addition, an elective practical course (subject area K; 112 teaching hours) must be taken during the main studies [34]. One of the five pharmaceutical core subjects (see below) can be chosen for this purpose. In seminars and practical exercises, a deeper insight shall be gained, e.g., into research tasks of the respective pharmaceutical sub-discipline.

At the end of the main study program, the second part of the pharmaceutical examination is due. The examination takes place in each of the five pharmaceutical core subjects, i.e., (a) pharmaceutical/medicinal chemistry, (b) pharmaceutical biology, (c) pharmaceutical technology/biopharmacy, (d) pharmacology and toxicology, as well as (e) clinical pharmacy [35]. An oral examination lasting 20–40 min is administered by a professor of the respective subject and university. The weighting among the five core subjects is again equal.

After this second pharmaceutical state examination, the training period at the university is actually completed. This is followed by a practical training year lasting a total of 12 months. At least 6 months of this must be spent at a community pharmacy. Such a public drug store represents the workplace of about three quarters of the prospective pharmacists (see below). It is also possible to pass the other 6 months, for example, in a hospital pharmacy, the pharmaceutical industry, or other institutions. The basic prerequisite is that pharmaceutical work is carried out on the one hand and that this is done under the supervision of a licensed pharmacist on the other [35]. This means that participation in pharmaceutical research at the university is possible. During the practical training year, seminars are given in preparation for the third pharmaceutical state examination. These are usually conducted in each state for two weeks per semester by the respective chamber of pharmacists. The contents are aimed at the examination syllabus. These include, on the one hand, pharmaceutical practice, and on the other hand, special areas of law for pharmacists. The examination is carried out by the government or chamber of pharmacists of the respective federal state. It is an oral examination, which should last at least 30 min and at most 60 min [35].

After successfully passing all three parts of the pharmaceutical state examination, an application can be submitted to the responsible authority in the federal state for licensure, the official professional authorization to practice as a pharmacist. The application includes the submission of several documents, such as a brief curriculum vitae, birth certificate, proof of health eligibility and criminal record and, of course, the certificates of the pharmaceutical state examination [39]. 

#### 3.1.4. Further Training Opportunities

During the entire course of pharmacy studies in Germany, no area of specialization is provided for. The elective practical course probably represents the only possibility to make a minimal focus on one of the five core subjects. It is generally a standardized course of study. The aim is to ensure that the education is as equivalent as possible in terms of content. The professional license already allows one to work as a pharmacist in all areas without additional specialization. Nevertheless, there is the possibility to gain further qualifications after graduation. In some professional fields, such continuing training is also desirable.

One option for further training in Germany is to acquire the title of specialist pharmacist. The training usually takes three years and includes participation in a number of seminars (120 h in total) offered by pharmacist chambers and the completion of a (practical) project. At the end, there is a final examination. There are different fields to become a specialist pharmacist. These include general pharmacy, clinical pharmacy, drug information, pharmaceutical analysis and technology, toxicology and ecology, theoretical and practical training, public health, as well as clinical chemistry ([40], pp.6–14).

In addition, a field of expertise can be acquired as a supplement or as an alternative. The training lasts one year and requires participation in seminars (100 h in total). The possible fields include naturopathy and homeopathy, nutrition counseling, geriatric pharmacy, prevention and health promotion, infectiology, medication management in the hospital, as well as nursing care ([40], pp. 17–22).

On top of these further education opportunities, which have a very strong connection to the different fields of activity in practice, there is also the possibility of gaining further scientific qualifications. At some locations (e.g., Fribourg, Greifswald, Halle-Wittenberg, Jena, Leipzig, Saarbrücken), it is possible to write a diploma thesis in pharmacy [41]. It is common to use six months of the practical training year to obtain the degree of diploma pharmacist. Moreover, some universities offer the possibility of obtaining a master’s degree in pharmaceutical research among other disciplines. The academic degree is a Master of Science (MSc) and usually requires four semesters to complete. There is also the option to pursue a doctoral degree or enroll in a PhD program to obtain the title of Dr. or PhD. This generally takes 3–5 years and can be performed at any of the 22 pharmacy university sites in Germany.

### 3.2. Pharmacy Studies in Vietnam

#### 3.2.1. Admission to Study

**Figure 3:**
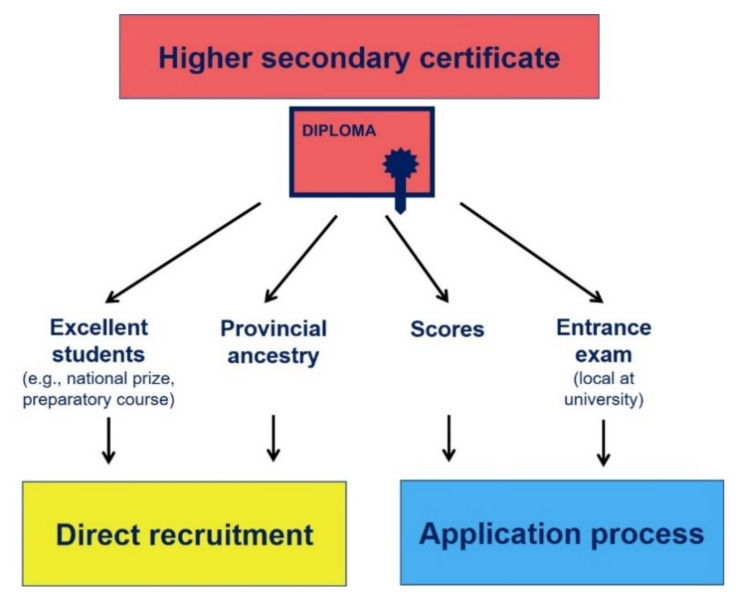
Flowchart of the various admission options to pharmacy studies in Vietnam.

In order to be able to study pharmacy in Vietnam, there are various ways to gain admission. Prospective students may be recruited or selected through an application process (see Figure 3). On the one hand, the criteria depend on the qualification (see below), with which the education is intended to be completed. On the other hand, individual aspects of the respective university also play a role in the context of getting access to studies.

The basic requirement is the higher secondary certificate. Students receive this certificate when they successfully pass the national higher secondary exam after 12 years at school. Excellent students in specialized classes or those who have received a national or international award in a science subject, for example, may even be recruited directly. This kind of straight access to studies also arises in cases where one comes from very provincial areas of the country and can demonstrate the quality of the registration documents. This option should also allow such pupils to study at the university. Family background, gender, and ethnic aspects can also come into play in this context [42]. Another example of direct enrollment may occur as a result of outstanding performance in a preparatory course.

The above-mentioned possibilities to get study admission, however, are not the most frequent. The most common way is to apply, which is mainly based on the scores in the high school certificate or, if applicable, in a local entrance examination provided by the respective university. They often represent thinking assessment exams, but aspects of science subjects are also covered. In order to successfully obtain a study place, a certain threshold regarding the score of the higher national secondary exam or the local entrance exam must be reached. The scores are individual for each university. However, apart from the total score, there may be thresholds for certain subjects, e.g., mathematics or chemistry, that must be achieved.

In addition to the general demand for pharmacists, pharmacy schools and universities can influence the setting of quotas for admission. Essential criteria for this are the respective capacities as well as factors that must be fulfilled to ensure high quality in teaching. The latter include, for example, the proportion of lecturers to the number of students as well as the space that can be given per student. However, these factors vary among the different locations [25].

#### 3.2.2. Study Locations

There are various pharmacy schools in Vietnam, but they differ in their competencies with regard to the degree they offer. Only at universities is it possible to obtain the grade of BPharm (bachelor of pharmacy), which is ultimately necessary to work as a "full" pharmacist. In addition, it is possible to acquire the lower-level degrees EDPharm (elementary diploma in pharmacy) and SDPharm (secondary diploma in pharmacy) as well as CDPharm (college diploma in pharmacy) at technical high schools and colleges, respectively, but not at universities. Postgraduate studies are exclusively possible at the universities.

Apart from military university, there are 16 public universities in Vietnam where pharmacy can be studied with a BPharm degree (see Table 2). There are an even greater number of private universities (2020: 18,150 pharmacy students), the number of which has increased in recent years. The establishment of pharmacy programs at private universities can be seen as a response to the growing demand for pharmacy professions. The number of pharmacy students in public universities in Vietnam was at total of 12,466 in 2020. In addition, the 16 public universities differ in which study programs are offered. Only in universities in the two largest cities of the country by far, the capital Hanoi in the north of the country and Ho Chi Minh City in the south, are all university-related degrees available. In this context, it should be mentioned that there are three study locations in Hanoi. However, in the capital, it is only the HUP that offers the full range of universal degrees. Tuition fees are generally required to study pharmacy in Vietnam at one of the above-mentioned locations. 

#### 3.2.3. Structure of the Study

**Figure 4:**
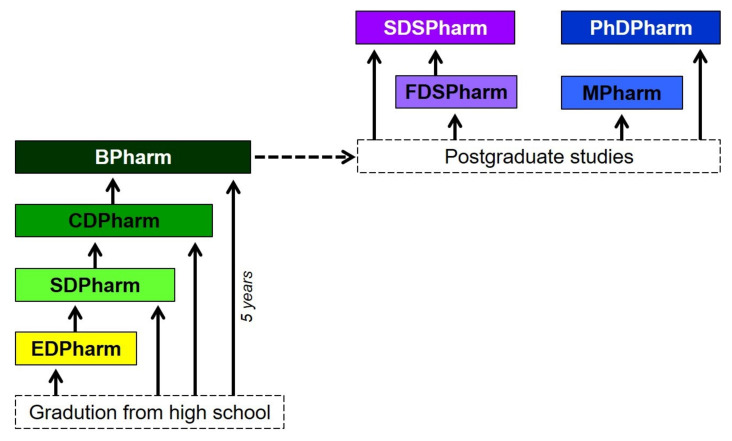
Overview of the various degrees that can be earned in pharmacy education in Vietnam.

When taking up pharmacy studies in Vietnam, there are some heterogeneous qualifications with which the studies can be completed (see Figure 4). The programs differ in the type and scope of content and consequently in the duration of study. Basically, pharmacy education at universities in Vietnam is based on national regulations, which are set by two ministries (Ministry of Education and Training, Ministry of Health). The guidelines include parameters such as the ratio of theoretical to practical courses and their corresponding credit points.

The EDPharm (elementary diploma in pharmacy) aims to acquire fundamental understanding and skills. It is characterized primarily by a high level of practical relevance. Students acquire competence to perform a limited number of simple tasks in daily pharmacy practice under the supervision of a fully qualified pharmacist. Such work includes, for example, obtaining information on medicinal products, making simple preparations, and advising on herbal medicines. The EDPharm training period lasts a total of one year, of which placements account for about one-fifth. However, obtaining the EDPharm is not very common in Vietnam because employment opportunities are severely limited.

The SDPharm (secondary diploma in pharmacy) lasts two years and is more focused on pharmaceutical knowledge. The training includes internships in the hospital pharmacy, pharmaceutical industry, quality control, and drug prescription among others. The principles of pharmacotherapy of diseases are to be learned through regular participation in a hospital ward.

The training for the CDPharm takes three years. It is characterized by more courses aiming at fundamental knowledge. In addition, students can select from optional modules. This approach is intended to enable them to identify and pursue their own interests in specific professional areas. Depending on the educational institute, different elective options are offered. Mandatory for all study sites, however, are courses on management and supply of drugs as well as their quality assurance.

**Figure 5:**
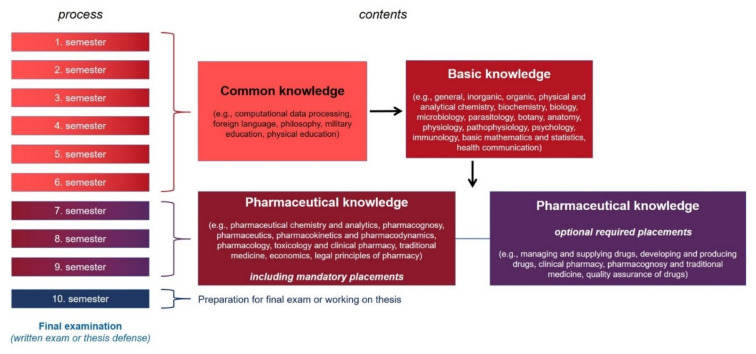
Schematic flow of the Vietnamese BPharm study program.

The degrees EDPharm, SDPharm, and CDPharm are inferior to the BPharm (bachelor of pharmacy). Nonetheless, after graduating with the degrees EDPharm, SDPharm, and CDPharm, students can go on to study BPharm. Achievements from the previous programs can be credited and the study time is thereby shortened individually (for a more detailed overview, a reference is made to literature [25]). The BPharm is necessary in order to be qualified to work as a full-fledged pharmacist in various professional fields. The BPharm program grants a broad education in pharmacy and the possibility of specializations. It lasts five years (10 semesters) and can be divided into three main units (see Figure 5): one for the acquisition of common knowledge, another part for basic knowledge relevant to understand the content of the third module, which is on deeper pharmaceutical subject matter.

To acquire common knowledge (HUP: 327 h for theory and 258 h for practice), the curriculum includes modules on, for example, data processing using computers, philosophical basics, foreign language, military education, or even sports. This part of the course is already part of the pharmacy curriculum. All undergraduate programs in Vietnam have such courses to train general skills and knowledge. The various courses for acquiring common knowledge are spread over several semesters. Usually, however, they are completed after the sixth semester.

A second major unit comprises courses designed to lay the foundations of natural science subjects, especially in chemistry, biology, and medical sub-subjects. Specifically, the courses address, for example, aspects of general, inorganic, organic, physical, and analytical chemistry as well as biochemistry. Other subjects include biology, microbiology, parasitology, and botany. Medical principles are covered in courses on anatomy and physiology, pathophysiology, psychology, and immunology. There are also classes on basic mathematics and statistics and health communication. The study section for the acquisition of basic knowledge at HUP comprises 368 h for theory and 127 h for practice. Foundation courses are typically taken prior to the beginning of the seventh semester, as students do not decide on and pursue a specialization until then.

Building on the foundational knowledge, in-depth pharmaceutical knowledge is subsequently taught and learned (HUP: 468 h for theory and 312 h for practice). Modules include pharmaceutical chemistry and analytics, pharmacognosy, pharmaceutics and industrial aspects, pharmacokinetics and pharmacodynamics, pharmacology, toxicology, and clinical pharmacy. Other modules focus on traditional medicine, economics, and legal principles of pharmacy.

The acquisition of special pharmaceutical knowledge also includes the completion of several placements. These take place in various health care and pharmaceutical settings. The aim is to gain an insight into the future professional work and to become familiar with the different professional environments. These practical trainings take place in parallel with the theoretical lectures and are concluded with an oral exam, a report, or a skills assessment.

In addition to placements in generalized pharmacy settings, the BPharm program also provides for specialized placements. Depending on the personal interest of the students and, if applicable, on the offers of the university, the students have to choose one out of five optional modules. Topics for these specialized placements cover (a) managing and supplying drugs (HUP: 194 h for theory and 76 h for practice), (b) developing and producing drugs (HUP: 210 h for theory and 90 h for practice), (c) clinical pharmacy (HUP: 115 h for theory and 115 h for practice), (d) pharmacognosy and traditional medicine (HUP: 210 h for theory and 60 h for practice), and (e) quality assurance of drugs (HUP: 218 h for theory and 52 h for practice). Thus, already during the course of studies, students have the opportunity to develop a specialization according to their own preferences.

At the end of the BPharm study program, a final examination is scheduled. This is done in written form. For some students who have consistently achieved very excellent scores throughout their studies, there is the opportunity to write a research dissertation and present the results of their thesis to a scientific board. The work for the thesis or the preparation for the final exam normally takes place in the last semester.

Depending on which of the various fields of activity pharmacists ultimately wish to work in, individual professional licenses must be applied for in Vietnam in each case. 

#### 3.2.4. Further Training Opportunities

After the BPharm, there is the possibility of postgraduate studies. However, these are only offered at three of the 16 public universities of pharmacy in Vietnam (see Table 2).

Basically, a distinction is made between two main systems. On the one hand, there is the so-called diploma of specialization FDSPharm (first-level diploma of specialization in pharmacy) and SDSPharm (second-level diploma of specialization in pharmacy). These represent the traditional type of postgraduate studies in Vietnam and are mainly aimed at generating practical knowledge and skills. On the other hand, there is a newer system with MPharm (master of pharmacy) and PhDPharm (doctor of philosophy in pharmacy). The latter two focus on competence for research and academia. A transfer between the traditional and the new model is in principle conceivable. After application for the program to be changed to and acceptance, the school checks the credits individually. In practice, however, this is so complex that the possibility of transferable accreditation between the two systems plays almost only a theoretical role.

The MPharm program lasts two years. While fundamental knowledge is taught and learned in the first year, the second year is dedicated to specialization in one of six different subjects. These are (a) pharmacognosy and traditional medicine, (b) pharmacology and clinical pharmacy, (c) toxicology and pharmaceutical analytics, (d) biochemistry, (e) pharmaceutical industry and pharmaceutics, as well as (f) pharmaceutical management and economics. There are a total of seven subjects for the PhDPharm. In addition to the subjects of the MPharm, there is also (g) pharmaceutical chemistry.

The MPharm degree and research experience are prerequisites for a PhDPharm, which in turn takes at least another three years. Excellent students can also enter the PhD studies directly after their graduation as a BPharm. However, they must complete MPharm courses in parallel.

The FDSPharm is similar to the MPharm, but is characterized by a stronger professional orientation. Therefore, more than half are practical courses. Finally, the SDSPharm is an extension of the FDSPharm. All four postgraduate studies are completed with a thesis.

## 4. Comparison

The general aim of a guest lectureship is to get to know the two education systems [43], to learn from each other, and finally to discover similarities and differences by comparing them (see Table 3). The comparison of BPharm studies in Vietnam with the pharmacy program in Germany was also implemented within the framework of the present perspective.

Germany is located in Central Europe and has about 83 million inhabitants. In terms of area, it is roughly comparable to Vietnam. The latter country is located in Southeast Asia and has a larger population of around 97 million. In both countries, there is a high demand for pharmacists [25,44], so their education plays an important role. It is also interesting to know what fields of employment the graduated pharmacists are working in.

In 2021, there were about 69,000 pharmacists working in Germany. Of these, the community pharmacy accounts for by far the largest share of over three quarters of all pharmacists (77.5%). This ranking is followed by employment in industry (11.4%) and hospitals (4.0%). Further areas of employment are universities (1.9%), authorities and corporations (1.7%), teaching institutions and schools (0.8%), and armed forces (0.3%), among others (2.3%) [45]. A survey at HUP of 2020 graduates in the year following graduation revealed that 93.5% found a job (after 3 months, 83.4% already had a job), while 3.5% pursued postgraduate studies (MPharm, PhDPharm). The remaining proportion had a job but gave it up at the time of the survey. The distribution among the different work areas was as follows: pharmaceutical representatives (26.5%), sales and distribution (22.8%), research centers (11.1%), hospital (8.3%), manufacture (6.4%), quality control (5.5%), regulatory affairs (4.9%), education (0.7%), government (0.7%), other fields (9.5%). The remaining proportion did not provide feedback during the survey (3.6%).

A fundamental difference between studying in both countries is the tuition fee. In Germany, there is no such fee, while in Vietnam, there is currently a VNĐ 24.5 million (Vietnamese Đong; equivalent to about USD 1000) per year (two semesters) tuition for pharmacy studies. However, tuition fees are to be gradually increased to VNĐ 35 million (about USD 1500) by the 2025/2026 academic year. The collection of fees is necessary because otherwise it would simply not be possible for the universities to offer high-quality studies. Especially in a country like Vietnam, where poverty still plays a role in the remote areas, the financial cost of the tuition fee (apart from the other living costs in the university cities) often excludes students from studying. Accordingly, the concept of basically free study at public universities for German students was very well received.

While in Germany, pharmacy can only be studied at universities, in Vietnam, there are different possibilities of pharmacy education: for example, in schools and colleges in addition to universities. Furthermore, the variety of pharmacy qualifications is more heterogenous in Vietnam. There are the EDPharm, SDPharm, and CDPharm. However, these are not sufficient for working as a full pharmacist. For this, the BPharm is required. Depending on the pharmaceutical field of activity, a separate professional license is required in each case. In Germany, one graduates as a pharmacist without an academic degree. After applying for the license, however, one can work as a pharmacist in all areas with this single professional authorization. This model appears more convenient for pharmacists.

There are some similarities in terms of access to studies. In both countries, access to pharmacy studies is limited and regulated. The grades in the high school diploma play a decisive role for admission, especially in Vietnam. There, students with very good higher secondary certificates can be recruited directly for the program. In Germany, too, the quota for the best school graduates gives excellent pupils an advantageous opportunity to obtain a place at university. This means that in both countries, particularly hard-working and high-achieving school graduates are supported with a view to obtaining a place at a pharmacy school.

By conducting local entrance examinations, prospective students in both countries are given the chance to increase their chances of obtaining a study place if they do well in these tests. These tests are similar in content, in that aspects from the same science subjects (mathematics, physics, biology, chemistry) are addressed, and they assess not only pure subject knowledge but also cognitive skills. 

Probably the most serious difference with regard to admission to studies is that in Germany, there is also the option to study at university without a certificate from the high school. By completing vocational training in the meantime, access to university is possible for a certain percentage. In Vietnam, on the other hand, the high school certificate or an equal certificate is absolutely mandatory.

In Germany, there are 22 locations to study pharmacy at a public university, while there are only 16 in Vietnam (see Table 1 and Table 2). In Germany, all of them are public universities, while in Vietnam, there are also private ones for studying pharmacy. The campuses in Germany are presumably distributed as evenly as possible across the country, which is not necessarily the case in Vietnam. In this context, however, the more uneven population density should also be considered as a reason.

The German universities of pharmacy differ in the possibility of starting pharmacy studies only once a year in the winter semester or even additionally in the summer semester. In Vietnam, on the contrary, the academic year provides only one single fixed time to start any study.

The various Vietnamese pharmacy degrees cannot be obtained equally at all universities there. At least in the past decade, there has been a successful effort to establish the BPharm system at all university campuses. There are also restrictions on where postgraduate studies can be carried out. This is only possible at three universities. In Germany, on the other hand, PhD studies can be undertaken without restriction at any of the 22 locations. The average duration of PhD studies appears to be the similar in both countries.

The postgraduate studies differ in both countries. Since there is no thematic specialization during the pharmacy studies in Germany, this can be done subsequently if interested. However, this is no longer carried out exclusively at the universities. There is the possibility of training to become a specialist pharmacist in various areas and/or further training in a field of expertise. However, these two further training options do not result in an academic degree. They are characterized in particular by the strong practical reference to a professional field. Thus, there are parallels to the FDSPharm and SDSPharm in Vietnam, which also have a strong application-oriented focus. However, in both these cases, the training is still provided at universities in Vietnam.

The contents and the formal procedure of the studies are regulated by national guidelines in both countries. In Germany, training to become a fully qualified pharmacist comprises four years of study at university and an additional year of practical training. The total duration of training is therefore five years. In Vietnam, too, it takes a whole of five years if you study directly to become a BPharm, the degree required to work in a pharmacy. While there are no real restrictions on total study time in Germany, students are rejected by Vietnamese universities after a maximum of eight years.

In both countries, the course of study can be divided into three parts. Although these are not completely identical, there exist clear parallels. In Vietnam, there is first a section that aims to generate common knowledge. Something comparable is not to be found in German pharmacy studies. This part is followed in Vietnam by a section to create basic knowledge. This part is very similar to the basic studies in Germany. The respective subjects are also very overlapping. The foundations are laid in chemistry, physics, biology, and medical-related fields. During this part of the studies, a placement is mandatory in Germany, which is supposed to give the students an insight into the professional processes working as a pharmacist. The analogous placements in Vietnam have the same purpose. There, however, they are more extensive and conclude with an assessment. In Germany, there is no explicit examination at the end of the internship.

Once the basics have been laid, the section follows in which in-depth pharmaceutical knowledge is taught and learned. This is identical in both countries and is referred to as main studies in Germany. There are clear similarities between the subjects. The five pharmaceutical core subjects in Germany can also be found in the Vietnamese program. In Vietnam, however, subjects such as economics and legal aspects are also included. In Germany, these are only taken up in the third part of the education, the so-called practical year. This includes mandatory work in the community pharmacy, which does not exist directly in the Vietnamese pharmacy studies. There, however, a large number of placements are mandatory during the phase for deepening pharmaceutical knowledge. These allow specialization during the course of study. This is not the case in Germany.

In Vietnam, the BPharm program is completed by a final exam. This is done in written form. In Germany, on the other hand, there are three main state examinations. While the first is written, the other two represent oral exams. In Vietnam, it is also possible for excellent students to write a thesis. This does not exist in Germany. At the moment, however, there is a fundamental discussion in Germany about changing pharmacy education. The introduction of a final thesis is a key point in the plans for change [46].

Another important aspect is the strengthening of the subject of clinical pharmacy. The purpose for this is that about three quarters of all graduates will work in public pharmacy, where pharmaceutical services such as patient care and pharmacotherapy play an essential role. The pharmacist’s job profile has changed from the manual production of drugs to their distribution to pharmaceutical care [47]. This change must also be kept pace with in university education. For years, efforts have been made in Germany to adapt university curricula to focus on clinical implementation, pharmacotherapy, and the patient herself/himself [48,49,50,51]. In Germany, the pharmacy curriculum is considered by critics to be overloaded with chemistry knowledge [38]. On the other hand, students consider the content of organic chemistry important for the understanding of drug action in medicinal chemistry and other subjects of the pharmacy curriculum [52].

This development is emerging in a similar way in Vietnam. Efforts are being made to promote the importance of clinical pharmacy in the future. Although it has not yet been given much consideration in the syllabus, the government is aware of its relevance. A strong clinical pharmacy fosters safety, effectiveness, and ultimately efficiency of pharmacotherapy, and finally, benefits society as a whole [25]. Therefore, the stronger implementation of the subject and its courses is also supported by ministries. Some of the inspiration comes from abroad, which shows that it is possible to learn from each other in this respect as well [25].

## 5. Conclusions

Germany and Vietnam are two countries of comparable size, but located on different continents. Of course, this causes cultural diversity and completely contrasting everyday life. However, one can be inspired by the other and get to know each other. This was the goal of a secondment of a pharmaceutical teaching employee from Germany to a Vietnamese pharmacy university. The stay was used to get to know the similarities and differences in the pharmacy education programs in both countries. While the basic structure of the pharmacy education programs in both countries is quite similar and of course show strong parallels especially in the content structure, there are also differences. These relate to the even stronger practical orientation in Vietnam and the option of specialization already during university training. In Germany, for example, specialization is possible after graduation if there is interest. The efforts in both countries to focus on the clinical application of drugs and the relationship to patients during the course of study demonstrate that a curriculum is not inflexible [53]. It can adapt to the possibly changing needs of society. There are good lessons to be learned from each other for implementing those changes in the context of such an evolution.

## Figures and Tables

**Table 1:** Geographically-ordered overview of the 22 pharmacy study sites in Germany (slightly modified from [34]).

Federal State	University	Start in Winter Semester	Start in Summer Semester
Baden- Wuerttemberg	Albert-Ludwigs-University Fribourg	✓	✕
	Eberhard-Karls-University Tübingen	✓	✕
	Ruprecht-Karls-University Heidelberg	✓	✕
Bavaria	Friedrich-Alexander-University Erlangen-Nuremberg	✓	✕
	Julius-Maximilians-University Wuerzburg	✓	✓
	Ludwig-Maximilians-University Munich	✓	✓
	University Regensburg	✓	✕
Berlin ^1^	Free University of Berlin	✓	✓
Brandenburg	- ^2^	-	-
Bremen ^1^	-	-	-
Hamburg ^1^	University Hamburg	✓	✕
Hesse	Johann-Wolfgang-Goethe-University Frankfurt	✓	✓
	Philipps-University Marburg	✓	✓
Lower Saxony	Technical University Braunschweig	✓	✓
Mecklenburg- Western Pomerania	University Greifswald	✓	✓
North Rhine- Westphalia	Heinrich-Heine-University Dusseldorf	✓	✓
	Rhenish Friedrich-Wilhelms-University Bonn	✓	✓
	Westphalian Wilhelms-University Muenster	✓	✓
Rhineland- Palatinate	Johannes-Gutenberg-University Mainz	✓	✓
Saarland	Saarland University Saarbrücken	✓	✕
Saxony	University Leipzig	✓	✕
Saxony-Anhalt	Martin-Luther-University Halle-Wittenberg	✓	✕
Schleswig-Holstein	Christian-Albrechts-University Kiel	✓	✓
Thuringia	Friedrich-Schiller-University Jena	✓	✕

^1^ Federal city state. ^2^ Plans on the establishment of a university pharmacy site.

**Table 2:** Alphabetical overview of the 16 public pharmacy university study sites in Vietnam.

University	Offered Degrees
Can Tho University of Medicine and Pharmacy	BPharm
Da Nang University	BPharm
Da Nang University of Medical Technology and Pharmacy	BPharm
Hai Phong University of Medicine and Pharmacy	BPharm
Hanoi University of Pharmacy	BPharm, MPharm, PhDPharm, FDSPharm, SDSPharm
Ho Chi Minh University of Medicine and Pharmacy	BPharm, MPharm, PhDPharm, FDSPharm, SDSPharm
Hue University of Medicine and Pharmacy	BPharm
National University Ho Chi Minh City	BPharm
Pham Ngoc Thach University of Medicine	BPharm
Tan Trao University	BPharm
Thai Binh University of Medicine and Pharmacy	BPharm
Thai Nguyen University of Medicine and Pharmacy	BPharm, FDSPharm (Clinical Pharmacy)
Tra Vinh University	BPharm
University of Medicine and Pharmacy, Hanoi National University	BPharm
Vietnam University of Traditional Medicine (Hanoi)	BPharm
Vinh Medicine University	BPharm

**Table 3:** Comparison of pharmacy studies in Germany and Vietnam (BPharm) based on some characteristic criteria.

Criterion	Germany	Vietnam
Demand for pharmacists	High	High
Tuition fee	None	Yes
Number of public universities offering pharmacy program	22	16
General admission	Limited, regulated	Limited, regulated
Access with excellent high school diploma	Special quota	Direct recruiting possible
Local aptitude test	Some few locations	All locations
Studying without high school diploma	Possible, if applicable	Impossible
Start of study	Normally one, some locations with two start dates	One fixed start date each year
Structure/content of study	Nationally regulated	Nationally regulated
Structure of education	Three-part (basic studies, main studies, training year)	Three-part (general knowledge, basic knowledge, pharmaceutical knowledge)
Interest in clinical pharmacy	Necessary, increasing	Necessary, increasing
Possibility of specialization	After graduation if interested	During acquiring pharmaceutical knowledge
Final exams	Three major exams (written, oral, oral)	One final exam (written or thesis)
Degree of undergraduate studies	No real degree	EDPharm, SDPharm, CDPharm, BPharm
Possibility of postgraduate studies	All pharmacy study sites	Limited to a few study sites
Credential to practice pharmacy	A single license for all fields of work	Different subjects depending on the field of work

## Data Availability

Not applicable.
